# HIV-1 CRF 02 AG polymerase genes in Southern Ghana are mosaics of different 02 AG strains and the protease gene cannot infer subtypes

**DOI:** 10.1186/1743-422X-6-27

**Published:** 2009-02-26

**Authors:** Kwamena W Sagoe, Magda Dwidar, Theophilus K Adiku, Max Q Arens

**Affiliations:** 1 Clinical Virology Laboratory, Department of Microbiology, University of Ghana Medical School, PO Box 4236, Accra, Ghana; 2 Retrovirus Laboratory, Department of Pediatrics, Washington University Medical School, St Louis, Missouri 63110, USA

## Abstract

**Background:**

Little is known about the detailed phylogeny relationships of CRF 02_AG HIV-1 polymerase genes in Ghana. The use of the protease gene of HIV-1 for subtyping has shown conflicting results.

**Methods:**

The partial polymerase gene sequences of 25 HIV-1 strains obtained with Viroseq reagents were aligned with reference subtypes and alignments trimmed to a 300 bp protease, 661 bp and 1005 reverse transcriptase sequence alignments. Phylogenetic relationships of these alignments were determined with the Neighbour-Joining method using 1000 replicates and recombination patterns determined for the sequences with RIP 3.0 in the HIV sequence database.

**Results:**

Unlike the other alignments, the protease gene had nodes with bootstrap values < 100% for repeat control sequences. Majority of the CRF 02_AG sequences from Ghana were made up of fragments of several strains of CRF 02_AG/AG strains. The protease gene alone is not suitable for phylogenetic analysis.

**Conclusion:**

The polymerase genes of HIV-1 strains from Ghana are made up of recombinants of several CRF 02_AG strains from Ghana, Senegal and Cameroon, but the clinical implications are unknown. Using the HIV-1 protease gene for subtyping will not infer subtypes correctly.

## Introduction

HIV-1 strains can be divided into three genetic groups (M, N and O) with the group M further divided into 9 pure subtypes [1-3]. Recombination has however led to the circulation of mosaic HIV-1 strains, and these include the circulation of circulating recombinant forms (CRF) which play an important role in the epidemic [4-9].

Several studies have used the polymerase (*pol*), protease (*prot.*), and reverse transcriptase (*RT*) genes for phylogeny [9-19]. Also, the *pol *gene has been shown to be useful for subtyping in areas with multiple subtypes [17]. In settings where the CRF 02_AG is found, fragments of the *RT *gene have been shown to provide a useful method for HIV-1 subtyping [9,12,14,15,17,18]. However, there are conflicting reports on the usefulness of the *prot. *gene for subtype classification [12,14,15,18].

In Ghana, the predominant subtype for the *prot. *gene is most likely to be CRF 02 AG [14]. Furthermore, it has recently been shown with HIV-1 envelope-glycoprotein gene (*env*-*gp41*) and *pol *sequences that most HIV-1 strains do not have strong phylogenetic relationships with each other [20,21], suggesting an extremely variable relationship between strains. Since the role of subtypes and recombinants in primary resistance to antiretroviral drugs is still evolving and therefore unclear, subtyping of all HIV-1 strains will be needed with resistance testing for patients failing therapy in countries with non-subtype B strains. With the scale-up of antiretroviral therapy in Ghana, there is an increased need to perform resistance testing for patients adhering to treatment, but still have elevated viral loads despite prolonged therapy. Since commercial kits like the ABI/Celera ViroSeq reagents (Celera Diagnostics, Foster City, CA) are expensive for drug resistance testing [11], the likelihood is that in-house assays will be developed for the *prot. *and partial *RT *regions and these fragments will also be used for subtype classification.

This study therefore determined the suitability of using the *prot*. and partial *RT *gene fragments of CRF 02_AG/AG-like sequences from Ghana which could be used for drug resistance testing, for subtype classification. The purity of the HIV-1 strains with respect to CRF 02_AG/AG-like strains involved in recombination were also looked at.

## Methods

### Sequencing of polymerase gene

Sequences from 25 patients infected with HIV-1 who attended the Fevers Unit at the Korle-Bu Teaching Hospital in Accra, Ghana, in 2003 were used for this study. The drug resistance mutations have been published recently [21]. Polymerase (*pol*) gene sequences were obtained using the ABI/Celera ViroSeq reagents (Celera Diagnostics, Foster City, CA) and this has been described elsewhere [11]. The nucleotide sequence data have been submitted to the NCBI database [GenBank: EF174555 to EF174569 and EF550529 to EF550538].

### Phylogenetic analysis

Sequence homology of the 25 sequences (GHN sequences) was done with the HIV Blast Search in the HIV sequence database  with a pair wise comparison. The sequences with the highest homology (n = 13) to the GHN sequences were aligned with HIV-1 reference subtypes and the 25 sequences obtained from Ghana using the Clustal W software in BioEdit version 5.0.6 .

Two of the sequences obtained from the Blast Search CRF 02_AG from Cameroon (MP569 [GenBank: AM279387]) and a subtype G from Nigeria (NG083 [GenBank: U88826]) were confirmed as already in the reference subtypes by a conservation plot using BioEdit. They were however included as internal controls (repeat sequences) for phylogeny. From this original alignment which was 1305 bp long (*pol.*), three additional files were created by trimming sequences so as to obtain alignments with different base lengths: 300 bp *prot*., 661 bp *RT *(*RT*s) and 1005 *RT*. The four alignments were exported in the Raw Text format to the PHYLIP software v3.66  and used for tree building. The *RT*s sequence includes amino acids 30 to 227 of the *RT *gene [22], and contains all the important drug resistance mutations for individual HIV-1 drugs currently being used in Ghana.

Distance estimations were done using Dnadist with the Kimura 2-parameter model [23], with the transition-to-transversion (T/S) ratios that built the best possible phylogenetic tree. The Neighbor-joining analysis was then used to create phylogenetic trees with 1000 datasets and trees rooted with an HIV-1 group O strain (MVP5180 [GenBank: L20571]). In order to build robust trees, SeqBoot was used to build 1000 replicates before distances were estimated. The T/S ratio was determined by using the Dnaml.exe file in the PHYLIP software to determine the maximum likelihood of obtaining the best tree. For each alignment (*pol*, *prot.*, *RTs*, and *RT*), the likelihood of having the best tree was determined by running the Dnaml.exe with a T/S ratio from 1 to 4 with incremental differences of 0.05. Since trees were going to be rooted with HIV-1 group O as an out-group, the MVP5180 strain was used as an out-group in Dnaml.exe for the T/S analysis. A consensus tree was built with Consense after Neighbor-joining and rooted with the MVP5180. Phylogenetic trees were displayed with the Molecular Evolutionary Genetics Analysis (MEGA) software version 4.0. Bootstrap values of 70% were considered as being phylogenetically significant.

### Recombination and CRF02_AG out-groups

Recombination analysis was done with RIP 3 in the HIV sequence database  with the 13 sequences obtained from the Blast Search as a background sequence alignment. After input of query sequences, the RIP 3 output was rerun to identify fragments of the GHN sequences which had high homologies to the sequences in the background sequence alignment. The window size for the analysis was set at 500 nucleotides because subtype inference for CRF 02_AG strains from Ghana have been done with a similar length of nucleotides [6]. The significant threshold for the RIP program was set at 90%.

Of the 25 GHN sequences, 22 were CRF 02_AG and 2 were unclassified. These 24 sequences were also aligned in a separate file and the T/S ratio for the best tree determined as described earlier. No out-group in Dnaml.exe was chosen for this T/S analysis and bootstrapping (1000 replicates) and Neighbor-Joining were used to infer phylogenetic relationships between the sequences. Trees were not rooted in Neigbor.exe (PHYLIP) and each sequence was subsequently used as an out-group and bootstrap values inferred in TreeView  after a consensus tree was built with Consense.

## Results

### Phylogenetic relationships

The T/S values for the likelihood of the best phylogenetic tree differed for each group of sequences analyzed. For the *pol*, *prot, RT*, and *RTs*, the values were 3.00, 1.85, 3.10 and 3.25 respectively. The file with the Ghana *pol *strains only had a T/S of 3.05.

**Figure 1:**
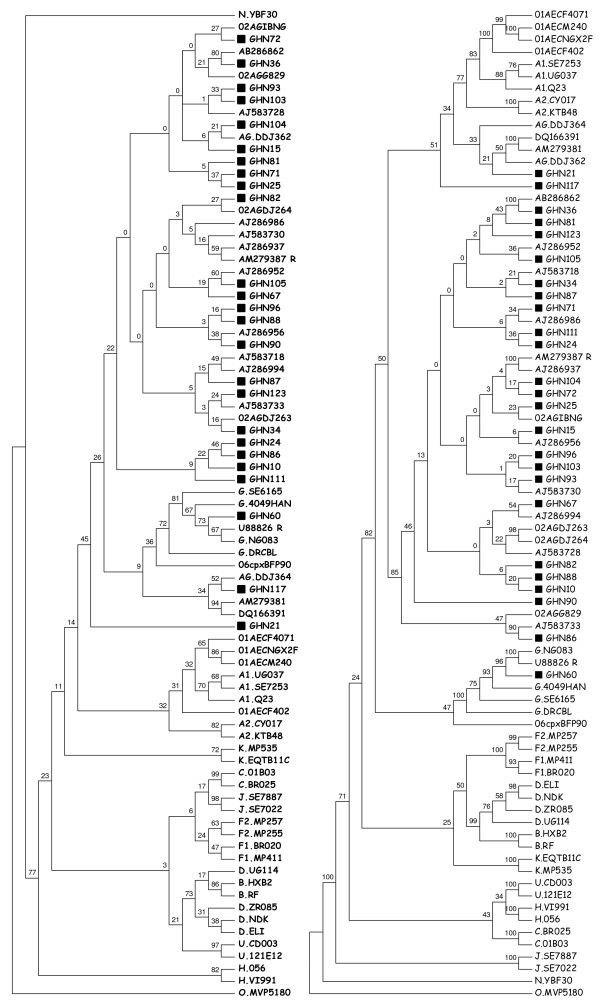
**Phylogenetic trees of sequences from 25 HIV-1 infected patients from Ghana showing relationships of the (a) 300 bp protease (left) and (b) 1305 bp partial polymerase genes (right)**. Reference subtypes have been prefixed; sequences with accession numbers are sequences obtained by HIV Blast Search. CRF 02_AG sequence with accession number AM279387_R and subtype G with accession number U88826_R are repeat sequences used as internal controls for phylogeny. The CRFs begin with 02 (CRF 02_AG), 01 (CRF 01_AE) and 06 (CRF 06_cpx). The sequences used in this study have been indicated by a filled square.

For the *pol*. and *RT*, GHN CRF 02_AG sequences were inferred with sufficient confidence (≥ 70%), but the *RTs *and *prot*. had bootstrap values of 57% and 22% respectively. Sequences which were repeated had 100% bootstrap values at their nodes for the *pol.*, *RT*, and *RTs*, but not the *prot*. Although the CRF 02_AG from Cameroon [GenBank: AM279387] and one of the reference subtypes [GenBank: AJ286937] were shown to have the same nucleotide sequences, the node for the two sequences had bootstrap value of 59% for the *prot*. alignment (Figure 1a). The subtype G from Nigeria [GenBank: U88826] that was repeated in the sequence alignment as U88826_R had bootstrap value of 67% for the *prot*. alignment (Figure 1a). The bootstrap values for the CRF 02_AG strains were 70% for *RT *and 57% for *RT*s, but their tree topologies were similar.

### Recombination patterns/Out-groups

Apart from GHN60, which was a subtype G, and GHN36 that was closely related to a CRF02_AG sequence from Ghana [GenBank: AB286862], all the GHN sequences were recombinants of various CRF 02_AG/AG-like strains from Ghana, Cameroon and Senegal [see Additional file 1]. One of the two unclassified strains (GHN21) was a recombinant of an AG recombinant (AG_CM [AM279381]) and a CRF 02_AG strain from Cameroon (02_AG CM [DQ166391]). The most frequent CRF02_AG fragments found were strains from Cameroon [GeneBank: AJ286952, AJ286956] and Senegal [GeneBank: AJ286994] [see Additional file 1]. Minor drug resistance mutations have recently being shown in the GHN sequences used in this study [21], but there was no obvious relationship between the nature of recombination and the mutations seen. Details of CRF02_AG/AG recombinant patterns for all sequences have been shown [see Additional file 1].

Even when considering a 90% homology of GHN sequences to those used as background in the RIP program, some level of recombination between CRF 02_AG and AG strains do occur. GHN36 and GHN60 were the only pure strains [see Additional file 1]. The phylogenetic relationships between the Ghana sequences alone showed that GHN90 and GHN21 (together with GHN117) were significantly presented as out-groups with very high bootstrap values (> 96%). None of the other sequences had significant bootstrap values as out-groups.

## Discussion

In this study, we trimmed sequences from a partial *pol *gene which included the *prot. *gene of HIV-1 sequences from Ghana. The results of this study unlike others presented the opportunity to determine phylogenetic relationships as the sequences were shortened from longer fragments and not by sequencing partial *pol *genes of the HIV-1 strains [15,17,18]. Our results indicate that the T/S values are different for different lengths of sequences and should be considered when building trees with fragments of the *pol *gene. The similarity in topology between *RT*s and *RT *shows that the 661 bp can be confidently used for subtyping of HIV-1 strains from Ghana.

Similar length of sequences in the *env *as compared to the *prot*. have been used to establish phylogenetic relationship in HIV-1 strains from Ghana [6]. This may mean that the variability in the *prot *gene, especially for CRFs, may not be sufficient to establish strain relationships. Our results for the *prot. *phylogeny are in contrast to that of others [12,14,15], but confirm the study by Pasquier et al [18]. The differences obtained from these studies are likely to be mainly due to the number of reference subtypes included. It is therefore important that in determining the true relationships of sequences, at least the nine pure subtypes and circulating recombinants commonly found within the region under study, are used for tree building. The subtyping done by Kinomoto et al. using only the *prot. *gene may therefore not be reliable [14].

The repeat sequences introduced had bootstrap values of 100% for the *pol*, *RT *and *RTs *phylogenetic trees but not *prot*. It can therefore be inferred that using a bootstrap value of 70% for the *RT *and 57% for the *RTs *which accounted for the CRF 02_AG cluster will be sufficient to determine subtypes. Although other studies have used higher values, our results indicate that it may be necessary to include repeat reference sequences in order to ascertain the reliability of the length of sequences being used for bootstrapping analysis. Since the repeat sequences in the *prot. *gene had bootstrap values < 100%, which did not reflect in the others, this test can be used as a standard to test for the reliability for HIV-1 phylogeny rather than arbitrarily fixing bootstrap values that support the confidence of relationships.

Our results confirm those of other studies that the *pol *and *RT *genes are useful for subtyping [17,18]. The loosely arranged *pol *gene sequences in the phylogenetic trees also reflected in the recombination analysis done, and confirm loosely arranged HIV-1 strains in previous studies [20]. Fragments of a previously characterized Ghanaian 02-AG sequence [GeneBank: AB286862 (4 in Additional file 1)] were found in only two sequences, GHN36 and GHN81 [see Additional file 1]. Since GHN36 was the only pure 02_AG strain found, this may suggest that the *pol *genes may have evolved away from this prototype into other sequences. The *pol *gene of 02_AG sequences may be undergoing complex recombination processes that may further complicate its use for subtyping. Furthermore, since GHN90 was clearly an out-group when the 24 Ghana sequences were analyzed alone, it is likely that the evolution is towards that strain. This may explain why fragments of CRF 02_AG strains [GeneBank: AJ286956 (5 in Additional file 1)] and [GeneBank: AJ583728 (7 in Additional file 1)] which were common in GHN90 were frequently seen in other GHN sequences [see Additional file 1].

Although GHN21 and GHN117 did not cluster with significant reliability with the AG recombinant reference sequences DDJ362 [GeneBank: AY521632] and DDJ364 [GeneBank: AY521633] even in the *pol *gene (Figure 1b), this can be explained with the recombination analysis done [see Additional file 1]. GHN21 and GHN117 both had fragments of CRF 02_AG strains in their sequences, with GHN117 having 5 as compared to one in GHN21 [see Additional file 1]. It will be impossible to make these inferences about the purity of GHN21 and GHN117, and the other GHN strains [see Additional file 1], without the RIP analysis.

Thus, the polymerase genes of HIV-1 strains from Ghana are made up of recombinants of several CRF 02_AG strains from Ghana, Senegal and Cameroon, but the clinical implications are unknown. A continuous surveillance of *pol *gene sequences from Ghana is needed to understand this evolutionary pattern.

## Competing interests

The authors declare that they have no competing interests.

## Authors' contributions

KWS, MD, TKA and MQA designed the study, acquired the data and analyzed the results. The authors were also responsible for writing the manuscript.

## Supplementary Material

Additional file 1 **Intra CRF 02_AG recombination patterns in the polymerase gene of HIV-1 strains from Southern Ghana. **ID_GHN _are the sequence numbers (Ghana sequences) which have GenBank accession numbers EF174555 to EF174569 and EF550529 to EF550538; **X **represents the presence of sequences 1 to 13 in a particular ID_GHN _strain; the strains and assertion numbers of sequences 1 to 13 are: 1 (CRF 02_AG or 02_AG CM [AJ286952]), 2 (02_AG SN [AJ286986]), 3 (02_AG CM, [AJ286937]), 4 (02_AG GH [AB286862]), 5 (02_AG CM [AJ286956]), 6 (02_AG SN [AJ583718]), 7 (02_AG SN [AJ583728]), 8 (02_AG SN [AJ583733]), 9 (02AG_SN [AJ583730]), 10 (recombinant AG_CM [AM279381]), 11 (02_AG CM [DQ166391]), 12 (subtype G NG [U88826]) and 13 (02_AG SN [AJ286994]); reference sequences 1 to 13 were obtained by using the Blast Search in the HIV database to identify the closest sequences to the 25 sequences from Ghana; ***T*_*CUM *_**is the cumulative occurrence of reference sequences 1 to 13 in all the 25 ID_GHN _sequences; ***T ***represents the number of times strains 1 to 13 are seen in recombinants; **R_COMB _**are recombination patterns in ID_GHN _using sequences 1 to 13 as background sequences in the HIV RIP 3.0 program in the HIV Sequence Database; **SS_90% _**represents stretches of nucleotides that had a homology of ≥ 90% in RIP analysis (stretches a large window sizes and may not necessarily be continuous); nil, no recombination. Click here for file
