# Supplementary material for: HIV-1 CRF 02 AG polymerase genes in Southern Ghana are mosaics of different 02 AG strains and the protease gene cannot infer subtypes
Source: Virol J. 2009 Feb 26;6:27. doi: 10.1186/1743-422X-6-27 (PMC2655287; doi:10.1186/1743-422X-6-27)
Supplement: Additional file 1 — Intra CRF 02_AG recombination patterns in the polymerase gene of HIV-1 strains from Southern Ghana. IDGHN are the sequence numbers (Ghana sequences) which have GenBank accession numbers EF174555 to EF174569 and EF550529 to EF550538; X represents the presence of sequences 1 to 13 in a particular IDGHN strain; the strains and assertion numbers of sequences 1 to 13 are: 1 (CRF 02_AG or 02_AG CM [AJ286952]), 2 (02_AG SN [AJ286986]), 3 (02_AG CM, [AJ286937]), 4 (02_AG GH [AB286862]), 5 (02_AG CM [AJ286956]), 6 (02_AG SN [AJ583718]), 7 (02_AG SN [AJ583728]), 8 (02_AG SN [AJ583733]), 9 (02AG_SN [AJ583730]), 10 (recombinant AG_CM [AM279381]), 11 (02_AG CM [DQ166391]), 12 (subtype G NG [U88826]) and 13 (02_AG SN [AJ286994]); reference sequences 1 to 13 were obtained by using the Blast Search in the HIV database to identify the closest sequences to the 25 sequences from Ghana; TCUM is the cumulative occurrence of reference sequences 1 to 13 in all the 25 IDGHN sequences; T represents the number of times strains 1 to 13 are seen in recombinants; RCOMB are recombination patterns in IDGHN using sequences 1 to 13 as background sequences in the HIV RIP 3.0 program in the HIV Sequence Database; SS90% represents stretches of nucleotides that had a homology of ≥ 90% in RIP analysis (stretches a large window sizes and may not necessarily be continuous); nil, no recombination. [file 1743-422X-6-27-S1.doc]

**Table 1**: Intra CRF 02_AG recombination patterns in the polymerase gene of HIV-1 strains from Southern Ghana

| ID_GHN_ | 1 | 2 | 3 | 4 | 5 | 6 | 7 | 8 | 9 | 10 | 11 | 12 | 13 | ***T*** | **R_COMB_** | **^♦^SS_90%_** |
| --- | --- | --- | --- | --- | --- | --- | --- | --- | --- | --- | --- | --- | --- | --- | --- | --- |
| 10 | X |  |  |  | X |  | X |  |  |  |  |  | X | 4 | 1/13/5/7 | 1 (1013-1022), 13 (466-582) |
| 15 | X |  |  |  | X |  | X |  |  |  |  |  |  | 3 | 5/1/5/7 | 5 (524-774) |
| 21 |  |  |  |  |  |  |  |  |  | X | X |  |  | 2 | 10/11 | 10 (250-747), 11 (783-1052) |
| 24 | X |  | X |  |  |  | X |  |  |  |  |  |  | 3 | 1/3/7 | 3 (459-860) |
| 25 | X |  | X |  | X |  | X |  |  |  |  |  | X | 5 | 3/1/13/5/7 | 7 (1008-1052) |
| 34 | X | X |  |  |  |  |  | X |  |  |  |  | X | 4 | 8/1/13/1/13/2/1 | 2 (710-924) |
| 36 |  |  |  | X |  |  |  |  |  |  |  |  |  | 1 | nil | 4 (250-1052) |
| 60 |  |  |  |  |  |  |  |  |  |  |  | X |  | 1 | nil | 12 (250-1052) |
| 67 |  |  |  |  | X | X |  |  |  |  |  |  | X | 3 | 13/5/6 | nil |
| 71 | X | X |  |  |  |  |  | X | X |  |  |  |  | 4 | 9/1/8/2/1/2/1/2/1 | 2 (832-872) |
| 72 |  |  | X |  | X | X | X |  | X |  |  |  | X | 6 | 3/13/5/9/7/6 | 6 (889-1052), 9 (837-840) |
| 81 | X | X |  | X | X |  |  |  |  |  |  |  | X | 5 | 4/1/13/5/2/1 | 5 (901-923) |
| 82 | X |  |  |  | X | X |  |  |  |  |  |  | X | 4 | 1/13/5/6 | nil |
| 86 | X |  | X |  |  |  |  | X |  |  |  |  |  | 3 | 1/3/8 | 8 (661-1052) |
| 87 | X | X |  |  | X | X |  |  |  |  |  |  | X | 5 | 1/13/1/5/2/6/5/2/6 | nil |
| 88 | X |  | X |  | X |  | X |  |  |  |  |  | X | 5 | 3/1/13/5/7 | 7 (1011-1022) |
| 90 | X |  | X |  | X |  | X |  |  |  |  |  | X | 5 | 5/1/13/3/7/5/7 | 5 (903), 7 (878-889), 13 (638-709) |
| 93 | X | X |  |  | X | X |  |  | X |  | X |  | X | 7 | 5/1/5/13/5/13/2/6/9/11 | 2 (663-874), 6 (899-961), 9 (971-1032), 11 (1040-1041) |
| 96 | X |  |  |  | X |  | X |  | X |  |  |  | X | 5 | 9/1/9/13/5/7/5 | nil |
| 103 | X |  | X |  | X | X | X |  |  |  |  |  | X | 6 | 3/1/3/13/5/6/7 | nil |
| 104 |  |  | X |  | X |  | X |  |  |  |  |  | X | 4 | 3/13/5/7 | 7 (1007-1052) |
| 105 | X |  |  |  | X | X |  |  |  |  |  |  | X | 4 | 1/13/5/6 | 1 (250-315), 5 (713-958) |
| 111 | X | X | X |  | X |  | X |  |  |  |  |  |  | 5 | 3/1/5/2/5/2/5/2/7 | nil |
| 117 | X |  | X |  |  | X | X | X |  | X |  |  |  | 6 | 10/3/1/6/8/7/10/7/10 | 1 (372-458), 6 (461-537), 7 (662-674; 740-792), 8 (564-661), 10 (677-732; 802-1052) |
| 123 | X | X |  |  | X |  |  |  |  |  |  |  | X | 4 | 13/5/13/1/13/5/2 | 2 (707-777) |
| ***T_CUM_*** | 19 | 7 | 10 | 2 | 17 | 8 | 12 | 4 | 4 | 2 | 2 | 1 | 16 | - |  |  |
